# Implementation Determinants of PrEP and Behavioral Health Treatment Referral among HIV Test Counselors

**DOI:** 10.1007/s10461-025-04620-2

**Published:** 2025-01-29

**Authors:** Audrey Harkness, Vanessa Morales, Kyle Grealis, Nequiel Reyes, Daniel J. Feaster, Steven Safren, DeAnne Turner, Raymond R. Balise

**Affiliations:** 1 School of Nursing & Health Studies, School of Nursing and Health Studies, University of Miami, 5030 Brunson Dr, Coral Gables, FL USA; 2 Department of Public Health Sciences, University of Miami School of Medicine, Miami, FL USA; 3 Department of Psychology, University of Miami, Coral Gables, FL USA; 4 College of Nursing, University of South Florida, Tampa, FL USA

**Keywords:** Implementation, HIV testing, Pre-exposure prophylaxis, Behavioral health, Referral, Implementación, Pruebas de VIH, Profilaxis previa a la exposición, Salud conductual, Referencia

## Abstract

Pre-exposure prophylaxis (PrEP), an effective biomedical prevention intervention, is not sufficiently reaching populations experiencing high HIV incidence. Behavioral health (BH) treatment addressing mental health and substance use similarly requires increased reach to HIV-affected populations. HIV testing is an opportunity to refer individuals to PrEP and BH treatment. This study, conducted in Miami-Dade County, FL, a domestic HIV epicenter, aimed to assess (1) self-reported rates at which HIV test counselors refer clients to PrEP and BH treatment, (2) barriers and facilitators to PrEP and BH treatment referral, and (3) the relationship between barriers and facilitators and test counselors’ referral rates. Among 127 HIV test counselors, the average PrEP referral rate was 63.8% (SD = 41.5) of those potentially meeting PrEP indications. Insufficient time was associated with lower PrEP referral (OR: 0.64, 95% CI: 0.42–0.99, p = 0.023) and training in PrEP screening was associated with higher rates of PrEP referral (OR: 1.27, 95% CI: 0.98–1.64, p = 0.034). The average BH treatment referral rate was 52.7% (SD = 44.4) of clients who the counselor felt would potentially benefit from treatment. Counselors lacking knowledge of screening (OR: 0.4, 95% CI: 0.2–0.78, p = 0.004), referral procedures (OR: 0.45, 95% CI: 0.23–0.87, p = 0.008), or locations to refer clients (OR: 0.47, 95% CI: 0.25–0.86, p = 0.008), as well as those with higher caseloads (OR: 0.998, 95% CI: 0.997–0.999, p < 0.001) were less likely to refer for BH. Training in substance use screening (OR: 1.26, 95% CI: 0.96–1.64, p = 0.046) and referral (OR: 1.28, 95% CI: 0.99–1.66, p = 0.029) were associated with increased BH referral. Implementation strategies are needed to address key barriers to PrEP and BH referrals in HIV testing contexts.

## Resumen

La profilaxis previa a la exposición (PrEP), una intervención de prevención biomédica efectiva, no está alcanzando adecuadamente a las poblaciones con alta incidencia de VIH. El tratamiento de salud conductual, que aborda la salud mental y el uso de sustancias, también necesita ampliar sus esfuerzos de alcance hacia las poblaciones afectadas por el VIH. Las pruebas de VIH son una oportunidad para referir a los individuos a PrEP y tratamiento de salud conductual. Este estudio, realizado en el condado de Miami-Dade, FL, un epicentro nacional del VIH, tuvo como objetivó evaluar (1) las tasas autoinformadas de referencia de los consejeros de pruebas de VIH a PrEP y tratamiento de salud conductual, (2) las barreras y los facilitadoras para la referencia a PrEP y tratamiento de salud conductual, y (3) la relación entre las barrearas y facilitadoras y las tasas de referencia de los consejeros de pruebas. Entre 127 consejeros de pruebas de VIH, la tasa promedio de referencia a PrEP fue del 63,8% (DE=41,5) de aquellos que potencialmente cumplían con las indicaciones para PrEP. El tiempo insuficiente se asoció con una menor referencia a PrEP (RM: 0.64, IC 95%: 0.42-0.99, p=0.023) y la formación en la detección de PrEP estuvo asociada con tasas mas altas de referencia a PrEP (RM: 1.27, IC 95%: 0.98-1.64, p=0.034). La tasa promedio de referencia al tratamiento de salud conductual fue del 52,7% (DE=44,4) de los clientes que el consejero considero que podrían beneficiarse del tratamiento. Los consejeros que carecían de conocimientos sobre la detección (RM: 0.4, IC 95%: 0.2-0.78, p=0.004), los procedimientos de referencia (RM: 0.45, IC 95%: 0.23-0.87, p=0.008) o los lugares para referir a los clientes (RM: 0.47, IC 95%: 0.25-0.86, p=0.008), así como aquellos con mayores cargas de trabajo (RM: 0.998, IC 95%: 0.997-0.999, p<0.001), tenían menos probabilidades de hacer referencias para tratamiento de salud conductual. La formación en la detección de uso de sustancias (RM: 1.26, IC 95%: 0.96-1.64, p=0.046) y en la referencia (RM: 1.28, IC 95%: 0.99-1.66, p=0.029) estuvo asociada con un aumento en las referencias a tratamiento de salud conductual. Se necesitan estrategias de implementación para abordar las barreras clave a las referencias de PrEP y tratamiento de salud conductual en los contextos de pruebas de VIH.

## Introduction

HIV continues to significantly impact communities across the United States (U.S.), despite availability of evidence-based prevention interventions that could end the epidemic [1, 2]. The U.S. Ending the HIV Epidemic (EHE) plan encourages scale-up and dissemination of pre-exposure prophylaxis (PrEP) to communities most affected by HIV [1]. PrEP is an effective biomedical intervention that prevents individuals exposed to HIV from acquiring it [3, 4], but reach to HIV affected communities remains suboptimal [5, 6]. In addition to PrEP, other evidence-based interventions, including behavioral health (BH) treatment (i.e., mental health and substance use treatment) also require scale-up and dissemination to HIV affected communities given evidence that BH concerns drive HIV incidence [7–10].

HIV test counselors are uniquely positioned to enhance the reach of both PrEP and BH treatment. People who seek HIV testing often do so following a potential exposure to HIV or concerns about acquiring HIV, making testing clients ideal PrEP candidates [11–14]. Testing clients may also have BH concerns, suggesting the utility of HIV test counselors being prepared to conduct BH treatment referrals [15]. However, this opportunity is frequently missed; prior research shows that only half of non-prescribing HIV prevention providers reported integrated PrEP screening in their organization’s HIV testing workflow and less than one-third screened their clients for PrEP referrals [16]. An evaluation of the CDC’s National HIV Prevention Program’s 2019 HIV testing data from non-healthcare settings showed that only about 47% of men who have sex with men (MSM) who were PrEP eligible were referred to PrEP by their HIV testers, with rates even lower for Latino and older MSM in the study [17]. PrEP referral rates have also been explored in the context of local health departments. Specifically, one study examined PrEP referral and prescribing at 85 local health departments in North Carolina, which serve a largely rural population [18]. Across these health departments, only 13% reported that they externally referred testing clients for PrEP and 4% reported that they prescribed PrEP on site. To our knowledge, research has not documented rates of BH treatment referral among HIV test counselors.

Limited prior research has examined determinants of HIV test counselors’ PrEP referrals, and even less has examined determinants of HIV test counselors’ BH treatment referrals. One qualitative study sheds light on some determinants, finding that lack of knowledge and self-efficacy were barriers to HIV test counselors referring for PrEP and BH treatment [19]. The complexity of making referrals was a particularly salient barrier to BH treatment referrals. These findings align with Turner and colleagues’ study which revealed qualitative differences in HIV test counselors’ knowledge, beliefs, and self-efficacy related to PrEP referral [20]. Mixed methods results revealed several factors within individual, organizational and extra-organizational contexts that influenced HIV test counselors’ PrEP referral practices, including available resources and the relative priority an organization put on referrals compared to other prevention tasks [21]. Additionally, HIV test counselors differentially discussed PrEP with clients based on their views of client eligibility and the availability of resources to guide the referral conversation [21]. Kundu and colleagues conducted a mixed methods study which identified barriers and facilitators to non-prescribing HIV prevention providers’ PrEP referrals, although these barriers and facilitators were not evaluated in relation to referral behaviors [16]. Their findings suggested that potential PrEP referral barriers at the provider level included lack of training on PrEP eligibility screening, concerns regarding PrEP stigma among clients, concerns that PrEP could cause harm if poorly implemented, lack of time, and lack of co-located PrEP services. Providers were also concerned that clients may not be able to access PrEP if referred (e.g., due to lack of insurance, financial instability, housing insecurity) and would stop using condoms if prescribed PrEP [16]. Providers in that study suggested that a variety of organizational factors, including peer support, continuing education, and training on PrEP could support their referral behaviors [16]. Another study examined PrEP referral implementation determinants at the organizational level only (i.e., reasons why local health departments in North Carolina did not provide external PrEP referrals), finding that referral implementation was impeded by a lack of local PrEP providers, lack of staff knowledge about PrEP, and perceptions that testing clients were not PrEP eligible [18].

Given the potential public health impact of HIV test counselors referring HIV testing clients to PrEP and BH treatment and building on our prior qualitative work, the current study sought to answer the following research questions: (1) what is the rate at which HIV test counselors in Miami-Dade County (MDC), an EHE priority jurisdiction, refer testing clients to PrEP and BH treatment?, (2) what are the most common referral barriers and facilitators?, and (3) which barriers and facilitators are associated with PrEP and BH treatment referral rates? Although some prior literature has examined referral rates for PrEP among HIV test counselors, the extant literature has not examined these referral rates specifically within Miami-Dade County, an EHE priority jurisdiction [1] or examined behavioral health treatment rates, to our knowledge. Similarly, although prior research has explored – qualitatively and quantitatively – endorsement of PrEP and behavioral health treatment referral barriers and facilitators, these determinants have not generally been linked to an outcome (i.e., referral rates). As such, the current study addresses important gaps in the extant literature.

## Methods

### Participants and Procedures

HIV test counselors completed surveys assessing their PrEP and BH treatment referral practices using REDCap [22]. Our inclusion criteria for the study were such that all HIV test counselors in Miami-Dade County who were certified by the Florida Department of Health were eligible to participate. We did not have selection criteria beyond this (e.g., there was not a limit on the number of counselors who could respond from the same organization). Participants were initially recruited in-person at test counselor re-certification training until COVID-19 at which point we shifted to flyers, email, social media, and snowball recruitment. Participants received ten dollars for completing surveys.^1^

## Measures

***Demographics***. Participants reported the number of years they worked as an HIV test counselor, the primary organization where they worked, and the estimated number of HIV tests they administered in the prior three months. To facilitate accuracy, the survey displayed participants’ corresponding weekly testing average based on their three-month estimate and allowed them to adjust their response.

***Referral rates***. Participants reported the number of their HIV testing clients who met PrEP indications and, of these, how many with whom they had “discussed and actively referred” to PrEP. To reduce social desirability bias, we included language normalizing that most HIV test counselors do not refer every client (i.e., “Many things can get in the way or make it harder to discuss and actively refer HIV testing clients to PrEP. Most test counselors don’t discuss and actively refer every client to PrEP.”). The PrEP referral rate was calculated as the number of PrEP referrals provided out of the total number of PrEP eligible clients (0–100%).

Participants were asked to indicate how many testing clients “could have benefitted from mental health or substance use treatment services,” and of those how many they actively referred to mental health or substance use treatment services. The BH referral rate was calculated as the number of BH referrals provided out of the total number of clients who could have benefitted from treatment (0–100%).

***Barriers to Referral Checklist***. We created two barriers to referral checklists, one for PrEP and one for BH treatment. The checklists were based on authors’ experiences providing HIV test counseling, research and training experiences with HIV test counselors, existing literature, and the CFIR (implementation determinants framework) [23, 24]. For the PrEP barriers to referral checklist, participants selected any response option that “made it harder in any way to discuss and actively refer clients to PrEP.” Example response options included, “The client was worried about side effects or the safety of PrEP,” “Not being trained to refer clients to PrEP,” and “Not having enough time during the HIV testing session.” All barriers are listed in Table 1.

**Table 1:** PrEP referral implementation determinants: descriptives and generalized estimating equations predicting odds of referral (N = 111)

CFIR^a^ Domain & Construct	Item	Endorsed N (%)	Generalized Estimating Equations OR When Predicting Referral (95% CI; *p*-value)	*p*-value
Innovation: Innovation Evidence-Base	Not having enough evidence that PrEP is effective*	3 (2.7%)	N/A **	N/A **
Innovation: Innovation Relative Advantage	Being concerned that the client would change their behavior as a result of being on PrEP*	5 (4.5%)	N/A **	N/A **
Inner Setting: Access to Knowledge and Information	Not being trained to refer clients to PrEP*	7 (6.3%)	N/A **	N/A **
	Not getting enough training to refer clients to PrEP*	4 (3.6%)	N/A **	N/A **
	Received formal training: motivational interviewing	67 (60.3%)	1.18 (0.94, 1.48)	0.074
	Received formal training: screening for PrEP eligibility	76 (68.5%)	**1.27 (0.98, 1.64)**	**0.034**
	Received formal training: completing a PrEP referral	68 (61.3%)	1.18 (0.93, 1.48)	0.085
Inner Setting: Compatibility	Not having enough time during the HIV testing session*	16 (14.4%)	**0.64 (0.42, 0.99)**	**0.023**
Inner Setting: Structural Characteristics – Work Infrastructure	The organization doesn’t have a policy for HIV test counselors to refer to PrEP*	1 (0.9%)	N/A **	N/A **
	Referring for PrEP is not a part of the HIV test counselor’s role*	1 (0.9%)	N/A **	N/A **
	Counselor caseload (i.e., estimated number of HIV tests counselor administered in prior three months)	Mean 85.5 (SD 145.2) Median 30 (Range 1–1000)	0.999 (0.998, 1.0)	0.113
Individuals: Innovation Deliverer – Other (Stigma)	Not wanting to offend the client by suggesting they should use PrEP*	4 (3.6%)	N/A **	N/A **
Individuals: Innovation Recipient—Other (Stigma)	The client seeming uncomfortable discussing PrEP*	18 (16.2%)	1.12 (0.9, 1.41)	0.156
Individuals: Innovation Recipients – Opportunity	The client didn’t think they would be able to pay for PrEP*	18 (16.2%)	1.15 (0.94, 1.43)	0.09
Individuals: Innovation Deliverer – Other (Assumptions and Biases)	Thinking the client wouldn’t be able to pay for PrEP*	6 (5.4%)	N/A **	N/A **
	Thinking that the client wouldn’t be interested or willing to use PrEP*	7 (6.3%)	N/A **	N/A **
Individuals: Innovation Deliverer – Other (Professional Experience)	Number of years counselor worked as an HIV test counselor	Mean 5.1 (SD 5.9) Median 3 (Range 0–39)	1.01 (0.995, 1.02)	0.101
Individuals: Innovation recipients – Capability	The client being concerned that they would change their behavior as a result of being on PrEP*	15 (13.5%)	0.87 (0.62, 1.22)	0.212
	The client didn’t think they would be able to adhere to PrEP*	22 (19.8%)	**1.26 (1.03, 1.55)**	**0.013**
Individuals: Innovation Recipients – Other (Concern about Side Effects of Intervention)	The client was worried about side effects or the safety of PrEP*	37 (33.3%)	1.11 (0.9, 1.35)	0.166
Individuals: Innovation Recipients–Need	The client saying they weren’t interested or willing to use PrEP*	46 (41.4%)	1.03 (0.84, 1.27)	0.391
	The client was already on PrEP*	20 (18.0%)	1.02 (0.77, 1.33)	0.457
Individuals: Innovation Deliverers – Capability	PrEP referral self-efficacy	Mean 3.1 (SD 1.4) Median 4 (Range 0–4)	1.07 (0.98, 1.16)	0.067
Other barrier	Something else*	10 (9.0%)	N/A **	N/A **
No barriers	There weren’t any barriers to PrEP referral*	26 (23.4%)	**1.29 (1.06, 1.57)**	**0.006**

Bold refers to predictors with a *p*-value < .05

^a^ Consolidated Framework for Implementation Science Research (CFIR)

^*^ These are items from the Barriers to PrEP Referral Checklist

^**^ These items were excluded from the GEE modeling

The BH barriers to referral checklist was structured the same way as the PrEP checklist. Participants were asked which barriers “made it harder in any way to discuss and actively refer clients to mental health or substance use treatment,” with options such as “Not knowing or being sure of how to screen for mental health/substance use concerns,” “Feeling uncomfortable discussing clients’ mental health/substance use,” and “The client saying they weren’t interested or willing to get treatment.” BH barriers are listed in Table 2.

**Table 2:** Behavioral health treatment referral implementation determinants: descriptives and generalized estimating equations predicting odds of referral (N = 104)

CFIR^a^ Domain & Construct	Item	Endorsed N (%)	Generalized Estimating Equations OR (95% CI)	*p*-value
Innovation: Innovation Evidence-Base	Thinking that MH/SU treatment wouldn’t be helpful or effective*	4 (3.8%)	N/A **	N/A **
Inner Setting: Access to Knowledge and Information	Not knowing or being sure of how to screen for mental health/substance use concerns*	19 (18.3%)	**0.4 (0.2, 0.78)**	**0.004**
	Not knowing or being sure of how to refer clients for mental health/substance use treatment*	13 (12.5%)	**0.45 (0.23, 0.87)**	**0.008**
	Received formal training: motivational interviewing	64 (61.5%)	1.06 (0.8, 1.4)	0.348
	Received formal training: screening for mental health concerns	46 (44.2%)	1.22 (0.93, 1.59)	0.074
	Received formal training: screening for substance use concerns	47 (45.2%)	**1.26 (0.96, 1.64)**	**0.046**
	Received formal training: completing a MH referral	36 (34.6%)	1.18 (0.91, 1.54)	0.11
	Received formal training: completing a SU referral	36 (34.6%)	**1.28 (0.99, 1.66)**	**0.029**
Inner Setting: Compatibility	Not having enough time to screen clients for MH/SU concerns*	12 (11.5%)	0.94 (0.61, 1.43)	0.379
	Not having enough time to refer clients for MH/SU treatment*	7 (6.7%)	N/A **	N/A **
Inner Setting: Structural Characteristics – Work Infrastructure	The organization doesn’t have a policy for HIV test counselors to refer to MH/SU treatment*	4 (3.9%)	N/A **	N/A **
	Referring to MH/SU treatment is not part of the HIV test counselors’ role*	9 (8.6%)	N/A **	N/A **
	Counselor caseload (i.e., estimated number of HIV tests counselor administered in prior three months)	Mean 80.7 (SD 139.8) Median 30 (Range 0, 1000)	**0.998 (0.997, 0.999)**	** < 0.001**
Individuals: Innovation Deliverer – Other (Stigma)	Not wanting to offend the client by suggesting they should receive MH/SU treatment*	12 (11.5%)	0.93 (0.58, 1.49)	0.374
Individuals: Innovation Deliverer—Capability	Feeling uncomfortable discussing clients’ MH/SU*	2 (1.9%)	N/A **	N/A **
Individuals: Innovation Deliverer – Other (Professional Experience)	Number of years counselor worked as an HIV test counselor	Mean 5.2 (SD 6.0) Median 3 (Range 0.5, 30)	1.01 (0.996, 1.03)	0.074
Individuals: Innovation Recipient—Other (Stigma)	The client seeming uncomfortable discussing their MH/SU*	35 (33.7%)	0.93 (0.69, 1.24)	0.302
Individuals: Innovation Recipients – Opportunity	The client didn’t think they could pay for treatment*	13 (12.5%)	0.76 (0.46, 1.26)	0.141
Individuals: Innovation Deliverer – Other (Assumptions and Biases)	Thinking the client wouldn’t be able to pay for treatment*	3 (2.9%)	N/A **	N/A **
	Thinking the client wouldn’t be interested or willing to get treatment*	6 (5.8%)	N/A **	N/A **
Individuals: Innovation Recipients—Need	The client saying they weren’t interested or willing to get treatment*	28 (26.9%)	0.76 (0.55, 1.07)	0.057
Individuals: Innovation deliverers – Capability	BH referral self-efficacy	Mean 2.2 (SD 1.7) Median 2.3 (Range 0, 4)	1.03 (0.95, 1.12)	0.257
	Not being sure if it was appropriate to refer clients for treatment*	15 (14.4%)	**0.45 (0.23, 0.88)**	**0.01**
Outer Setting: Partnerships & Connections	Not knowing or being sure of where to refer clients for treatment*	16 (15.4%)	**0.47 (0.25, 0.86)**	**0.008**
Other barrier	Something else*	6 (5.8%)	N/A **	N/A **
No barriers	There weren’t any barriers to BH referral*	26 (25%)	**1.47 (1.16, 1.85)**	** < 0.001**

Bold refers to predictors with a *p*-value < .05

^a^ Consolidated Framework for Implementation Science Reserach (CFIR)

^*^ These are items from the Barriers to Behavioral Health Treatment Referral Checklist

^**^ These items were excluded from the GEE modeling

***Self-efficacy***. Participants rated their self-efficacy to engage in referral behaviors from 1 (not confident at all) to 4 (completely confident). Three items were averaged to create a PrEP referral self-efficacy score (α = 0.88). These items included “Have a brief conversation with HIV testing clients about PrEP,” “Assess a client’s eligibility for PrEP,” and “Motivate a client to seek PrEP.” Six items were averaged to create a BH referral self-efficacy score (α = 0.95). Sample items included, “Have a brief conversation with HIV testing clients about their mental health,” “Assess an HIV testing client’s substance use treatment needs,” and “Motivate a client to seek mental health treatment.

***Training history***. Participants were asked whether they received formal training in: motivational interviewing, PrEP eligibility screening, mental health screening, substance use screening, referring for PrEP, referring for mental health treatment, and referring for substance use treatment. These were treated as a single-item measures, depicted in Tables 1 and 2 (“inner setting: access to knowledge and information” row).

***Implementation Strategies to Improve Referrals***. Participants were presented with a checklist of implementation strategies that could increase their PrEP or BH referrals. Examples included, “Having a protocol to follow to help me assess for eligibility/need for services,” “Having meetings with other HIV test counselors to learn about referral techniques and processes,” and “Having a directory of available providers to whom I can make referrals.” All items are presented in Table 3.

**Table 3:** Implementation strategies endorsed as potentially helpful for increasing prep and/or behavioral health treatment referral by HIV test counselors (N = 127)

ERIC^a^ taxonomy	Item from Survey	Frequency Endorsed N (%)	Examples of How the Strategy Could Address Significant or Frequent Implementation Barriers
Develop a formal implementation blueprint	Having a protocol to follow to help me assess for eligibility/need for services	58 (45.7%)	Could address the significant behavioral health referral barriers of not knowing how to screen for mental health/substance use concerns and not being sure if it was appropriate to refer clients for treatment
	Having a protocol to help me refer out for services	45 (35.4%)	Could address the significant behavioral health referral barrier of not knowing how to refer
Promote network weaving	Having a directory available of providers to whom I can make referrals	56 (44.1%)	Could address the significant behavioral health referral barrier of not knowing where to refer clients for treatment
Create a learning collaborative	Having meetings with other HIV test counselors to learn about referral techniques and processes	54 (42.5%)	Could address frequently endorsed PrEP referral barriers (e.g., client concerns about side effects, lack of client interest) by discussing in these meetings how other counselors have addressed these client concerns when referring
Conduct ongoing training	Getting ongoing training from an expert in the referral process	51 (40.2%)	Could address frequently endorsed PrEP and behavioral health referral of client seeming uncomfortable if the training specifically addresses how to broach uncomfortable topics with clients
Provide ongoing consultation	Having a phone number I can call or text to guide me/my client through the referral process	41 (32.3%)	Could address significant behavioral health referral barrier of not knowing how to refer or where to refer by reducing the amount of information the HIV test counselor needs to have on hand to make a referral
Identify and prepare champions	Having a designated person at my agency who leads the referral process	38 (29.9%)	Could address multiple knowledge-related barriers by re-assigning the referral role to a non-HIV test counselor
Conduct educational outreach visits	Getting a one-time training from an expert in the referral process	36 (28.3%)	Could address multiple knowledge-related barriers by increasing HIV test counselors’ knowledge about the referral process
Revise professional roles	Having additional time during HIV testing sessions to assess needs and make referrals	26 (20.5%)	Could address the significant PrEP referral barrier of not having enough time during testing sessions by providing HIV test counselors with more flexibility during sessions

^a^Expert Taxonomy for Implementing Change (ERIC)

^*^Here, we only consider determinants that were observed as significantly associated with referral rates; strategies may also address other determinants

## Analytic Approach

First, we mapped all barriers and facilitators onto the CFIR 2.0 [24] (Tables 1 and 2). We also mapped the implementation strategies onto the ERIC implementation strategy taxonomy [25] (Table 3).

Next, we used graphical and numerical exploratory data analysis methods to assess HIV test counselors’ referral rates and identify the most frequently endorsed barriers to each type of referral.

We used Generalized Estimating Equations (GEE) methods to evaluate which barriers/facilitators were associated with counselors’ referral rates. GEE models accounted for clustering of client-level referral data within HIV test counselors and assessed the relative increased or decreased odds of referring as a function of yes and no responses to the barriers/facilitators. Any predictors endorsed by < 10 participants were excluded from GEE models to improve stability of estimates. Prior to modeling, we removed participants for two non-mutually exclusive reasons: (1) participant reported not testing any clients in the past three months (N_PrEP_ = 11, N_BH_ = 18) and (2) participant reported referral rates that were inconsistent with their testing rates (i.e., those who reported referring more clients than they tested; N_PrEP_ = 6, N_BH_ = 8). To illustrate the relationship between significant predictors and referral rates, we created multi-panel histogram plots, which depict participants’ responses to each significant predictor. These plots were augmented to show the average proportion of clients who were or were not referred as a function of barriers/facilitators.

All analyses were conducted using R version 4.3.2 with the rUM (v1.0.2) [26], tidyREDCap (v1.1.1) [27] and portions of the tidyverse meta package (v2.0) [28] used to import, process and visualize the data. GEE analyses were conducted using the gee package (v4.13.26). All hypothesis tests were two-tailed and results p < 0.05 considered statistically significant.

## Results

### Descriptive Statistics: Referral Determinants

**Fig. 1:**
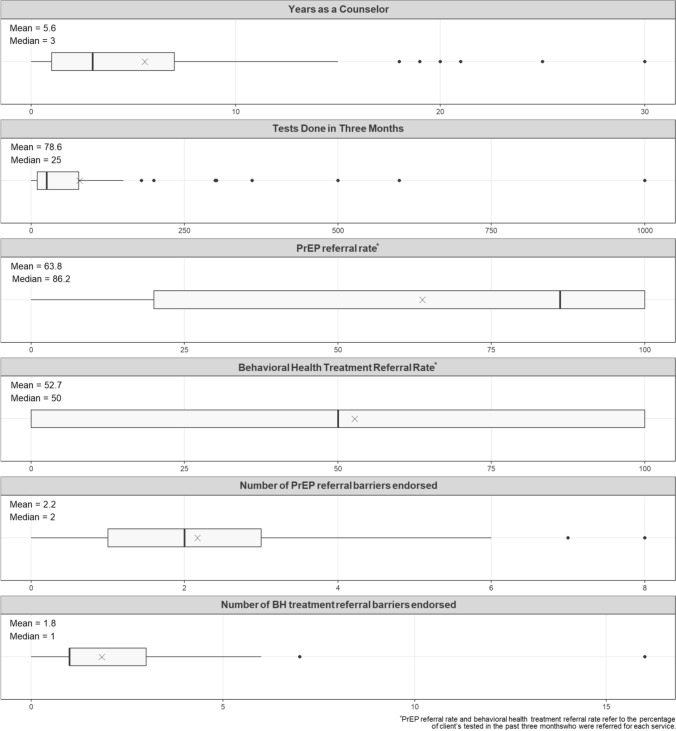
Participant demographic and descriptive information (N = 127)

HIV test counselors’ (N = 127) demographic and descriptive characteristics varied substantially (*Fig. 1). In total, counselors represented 31 different organizations. Typically, each organization had 1 (mode) counselor represented in the current study’s sample, with the number of counselors per organization ranging from 1 to 13 (25th percentile: 1, 75th percentile: 5. The volume of testing done varied greatly across counselors. The median number of tests administered was 25 but the average was 78.6 due to counselors who tested hundreds of clients. The median referral rate for PrEP was 86.2% but the average was 63.8% due to counselors who referred rarely. The mean and median BH treatment referral rate was approximately 50%.

Typically, counselors endorsed two PrEP referral barriers, but some counselors experienced many more, including one counselor who reported eight PrEP referral barriers. Compared to PrEP, there were fewer BH treatment referral barriers (median of 1), with the mean being higher due to a counselor who reported 17 barriers. The five most common PrEP referral barriers (*Fig. 1) were “The client saying they weren’t interested or willing to use PrEP” (41.4%), “The client was worried about side effects/safety of PrEP” (33.3%), “The client didn’t think they would be able to adhere to PrEP” (19.8%), “The client was already on PrEP” (18.0%) and tied for the fifth most common barrier were: “The client seeming uncomfortable discussing PrEP” (16.2%) and “The client didn’t think they would be able to pay for PrEP (16.2%). The most commonly endorsed BH treatment referral barriers were: “The client seeming uncomfortable discussing their mental health/substance use” (33.7%), “The client saying they weren’t interested or willing to get treatment" (26.9%), “Not knowing/being sure how to screen for mental health/substance use concerns” (18.3%), “Not knowing/being sure where to refer clients for treatment” (15.4%) and “Not knowing/being sure how to refer clients for mental health/substance use treatment” (12.5%).

## Predictors of Referral Rates

**Fig. 2:**
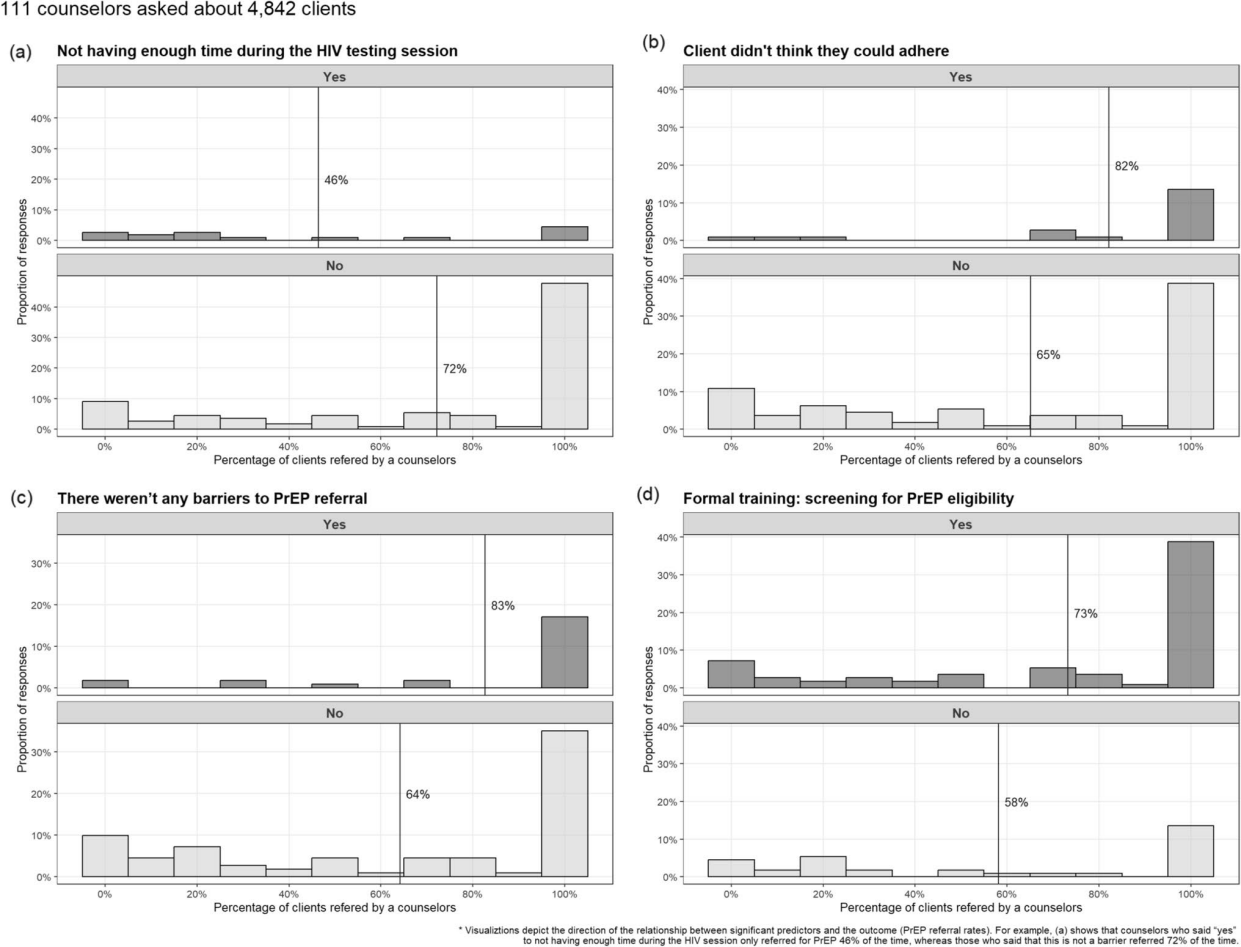
Significant GEE predictors of PrEP referral

GEE models were used to first examine the relationship between the PrEP barriers/facilitators and referral rates. The analytic sample was 111 counselors who reported a total of 4,842 testing clients in the past 3 months. Four determinants were associated with counselors’ PrEP referral rates (Table 1 and Fig. 2). A barrier associated with reduced odds of PrEP referral was “Not having enough time during the HIV testing session” (OR: 0.64, 95% CI: 0.42–0.99, p = 0.023). Unexpectedly, one barrier, “Client didn’t think they would be able to adhere to PrEP” (OR: 1.26 95% CI: 1.03–1.55, p = 0.013) was associated with increased odds of PrEP referral. The absence of any barriers was associated with increased odds of PrEP referral (OR: 1.29, 95% CI: 1.06–1.57, p = 0.006). Finally, a facilitator “Received formal training: screening for PrEP eligibility” was associated with greater odds of PrEP referral (OR: 1.27, 95% CI: 0.98–1.64, p = 0.034). Figure 2 visualizes the relationship between the significant determinants and referral rates, also showing how common it was to make a referral or not based on endorsement of each predictor. For instance, counselors who said they did not have enough time only referred for PrEP 46% of the time, whereas counselors who did not endorse this barrier referred 72% of the time.

**Fig. 3:**
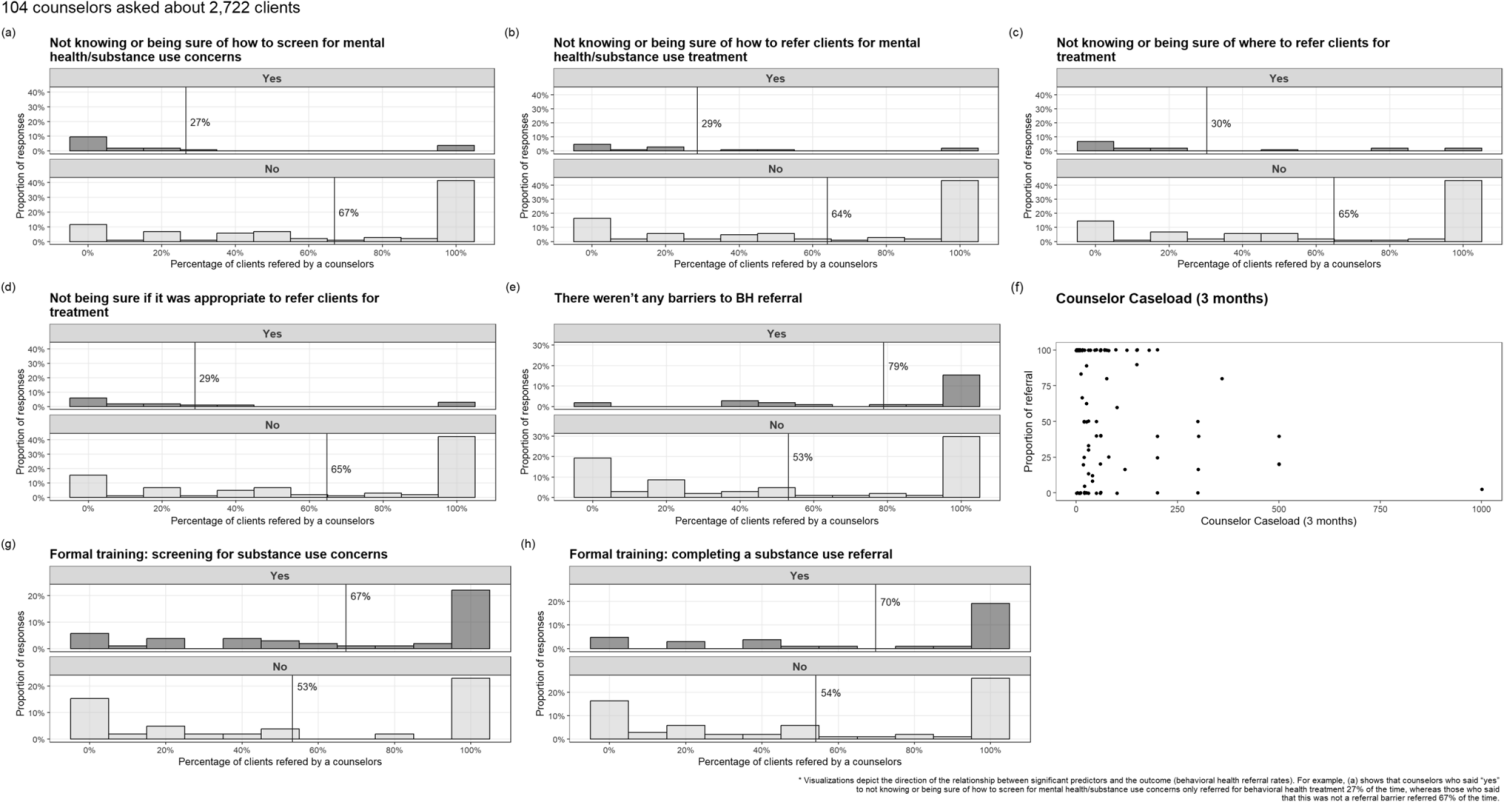
Significant GEE predictors of behavioral and substance use counseling

The second set of GEE models assessed if BH barriers/facilitators were associated with referral rates. The analytic sample was 104 counselors who reported a total of 2,722 testing clients in the past three months. These models identified eight determinants that were significantly associated with BH referrals. Several barriers were associated with reduced odds of referring, including: “Not knowing/being sure how to screen for mental health/substance use concerns” (OR: 0.4, 95% CI: 0.2–0.78, p = 0.004), “Not knowing/being sure how to refer clients for mental health/substance use treatment” (OR: 0.45, 95% CI: 0.23–0.87, p = 0.008), “Not being sure if it was appropriate to refer clients for treatment” (OR: 0.45, 95% CI: 0.23–0.88, p = 0.01), and “Not knowing/being sure where to refer clients for treatment” (OR: 0.47, 95% CI: 0.25–0.86, p = 0.008). Counselors who had higher caseloads (i.e., reported a higher volume of testing sessions in the past three months) had lower odds of referral (OR: 0.998, 95% CI: 0.997–0.999, p < 0.001). In contrast, counselors who received formal training in screening for substance use concerns (OR: 1.26, 95% CI: 0.96–1.64, p = 0.046) or completing a substance use referral (OR: 1.28, 95% CI: 0.99–1.66, p = 0.029) had greater odds of referring. Counselors who reported a lack of any barriers to BH referrals were more likely to refer (OR: 1.47, 95% CI: 1.16–1.85, p < 0.001). Figure 3 visualizes the relationship between the significant determinants and BH treatment referral rates and shows how common it was to make a referral or not based on endorsement of each predictor. For instance, counselors who said they did not know how to screen for BH concerns only referred for treatment 27% of the time, whereas those who did not endorse this barrier referred 67% of the time.

## Implementation Strategies

Finally, we examined which implementation strategies counselors believed would be most helpful for increasing their rates of PrEP and BH treatment referral (Table 3). Developing a formal implementation blueprint, operationalized as “having a protocol to follow to help me assess for eligibility/need for services,” was the most endorsed strategy (N = 58, 45.7%). Promoting network weaving, operationalized as “Having a directory available of providers to whom I can make referrals” was the next most endorsed strategy (N = 56, 44.1%). The third most endorsed strategy was creating a learning collaborative, operationalized as “Having meetings with other HIV test counselors to learn about referral techniques and processes,” (N = 54, 42.5%). Table 3 provides illustrative examples of how each strategy could address significant or frequent implementation barriers identified in the current study.

## Discussion

This study showed there are opportunities to increase HIV test counselors’ referral rates for PrEP and BH treatment. Further, we evaluated the frequency of referral barriers and facilitators for PrEP and BH, and their association with referral rates. The key barrier to PrEP referral was insufficient time, whereas significant BH referral barriers were not knowing how to screen, how to refer, and where to refer, uncertainty about the appropriateness of referring HIV testing clients for BH, and having a high testing caseload. Findings suggest the need for implementation strategies to address implementation barriers to increase referral rates for both types of services, given that HIV testing is an ideal context for referring based on the needs of individuals presenting for testing [15] and due to the link between BH and HIV [9, 29].

On average, HIV test counselors referred their clients to PrEP about two-thirds of the time (median = 86.2%). Although counselors referred more often than not, opportunities to increase referrals remain. Our findings showed that some implementation determinants are associated with PrEP referral rates, and therefore may be useful targets when developing PrEP referral implementation strategies. Specifically, we found one barrier associated with lower rates of PrEP referral: insufficient time during the testing session. This is consistent with our prior qualitative work showing that PrEP referrals can be complex and time consuming, impeding referrals [19]. Others have also shown that time constraints and high client flow can be a challenge for implementing innovations among HIV test counselors [16]. Researchers have highlighted STI clinics as a potential setting for implementing PrEP, given their high patient volume, focus on sexual health, and ongoing engagement with populations who would benefit from PrEP; however, they also cite limited counseling time as a potential barrier to implementation in this setting [30]. Time as a referral barrier reflects the need for an implementation strategy that targets the inner setting (i.e., organizations where counselors work) to reduce the impact of time constraints on referrals. Although one option could be to increase the time allotted for testing, this may not be feasible given organizational capacity. Alternative strategies, such as creating efficient protocols, creating a referral directory, or outsourcing to a referral specialist may be other ways of addressing this barrier. Technology-based tools that help counselors assess client need for PrEP and facilitate referral may be particularly promising for addressing this barrier. Several such tools exist or are in development, including LYNX (a mobile application to help link MSM HIV testing clients to PrEP);[30], MyChoices (a mobile application to increase HIV testing and PrEP uptake among MSM); [31], HealthMindr (a mobile application based on social cognitive theory to promote several health outcomes including PrEP uptake); [32], and the JUNTOS Referral Network (a website tailored to Latino MSM to improve referral to and uptake of PrEP and other HIV and ancillary services) [33]. Although bringing these tools into HIV testing contexts may bring new implementation challenges, they may mitigate time as a barrier to PrEP referral, which could help to avert new HIV diagnoses given the volume of clients who come through HIV testing contexts and may benefit from PrEP.

We also identified determinants associated with higher PrEP referral rates. Receiving formal training was an inner setting determinant associated with more PrEP referrals. Training HIV test counselors in PrEP referral processes may be a useful strategy for increasing referral rates. This is consistent with our prior qualitative work, which suggested the need for additional training in PrEP referrals and potential benefits of offering PrEP navigator training to HIV test counselors [19]. Combining our prior work with our current findings suggests that expanding access to PrEP navigator training to all HIV test counselors could be another implementation strategy to improve PrEP referrals, particularly in settings where PrEP is not offered on site (i.e., the process of referring is likely more complex than in a setting where PrEP is available within a neighboring department in the same site). Researchers have identified the “purview paradox” as a barrier to implementing PrEP in healthcare settings; specifically, that HIV specialists who are knowledgeable about PrEP do not tend to see HIV-negative patients, whereas primary care providers frequently encounter HIV-negative patients who might benefit from PrEP but are not sufficiently trained in or knowledgeable about PrEP [34, 35]. It may be that in the context of HIV testing, a different type of purview paradox could be occurring, whereby counselors may be ideally situated to identify PrEP candidates but need more formal training to do so, whereas PrEP navigators are most able to answer detailed questions about PrEP but only see those already interested in PrEP services.

In this study, counselors referred to BH treatment substantially less often than they referred to PrEP. BH referral barriers associated with fewer referrals included: 1) lack of knowledge about screening and referral processes, 2) uncertainty about the appropriateness of referring for BH, and 3) lack of referral partnerships for BH treatment; many of which were consistent with our prior qualitative findings [19]. We also found that those with higher caseloads were less likely to make referrals. High caseload has been identified as a barrier to implementing other evidence based interventions because of job stress/burnout, paperwork/documentation, pressure to meet quotas, and insufficient time to complete evidence based intervention training due to caseload [36–38]. Our study fills key gaps in the extant literature regarding BH referral in the context of HIV testing, as no prior quantitative work to our knowledge has examined implementation determinants in this area. Referral for BH treatment may be particularly important for HIV prevention due to the relationship between BH and sexual behavior that can lead to HIV acquisition [8, 39], and PrEP non-adherence [40, 41]. Our findings suggest that developing a formal implementation blueprint that guides counselors to assess and refer for services, having a referral directory, building provider connections, and reducing counselor caseload could help to address these barriers. Although there is increasing interest and movement toward co-locating BH services in HIV treatment settings [8, 42, 43], BH treatment may not be as commonly available at HIV testing sites. HIV test counselors may therefore be particularly likely to benefit from forming strong referral relationships outside their own organization and having an up-to-date referral directory to enable their referrals to BH treatment.

We also identified facilitators associated with higher rates of BH referral. Substance use screening and referral training were each associated with higher likelihood of BH referral. Although participants endorsed receiving mental health screening and referral training at similar rates as substance use screening and referral training, endorsement of these was not associated with higher referral rates. Our prior qualitative work suggested it was easier for counselors to refer for substance use than mental health because substance use screening is standardized in the HIV testing workflow via a form that all Florida HIV test counselors are required to use [19]. Accordingly, substance use screening/referral training may have been more compatible with existing referral resources than the mental health screening/referral training. Mental health screening and referral training may be insufficient to promote counselor referral to mental health treatment without specific screening tools that are integrated into their workflows. As such, adding brief mental health screening assessments to existing HIV testing workflows – as is done in other healthcare settings (e.g., depression screening in primary care for pregnant and postpartum individuals [44], PTSD screening in primary care with veterans [45]) – might help counselors improve their referral rates when combined with mental health screening and referral training.

We also had a counterintuitive finding. One PrEP barrier was associated with more PrEP referrals: the client thinking they would not be able to adhere to PrEP. As a cross-sectional study, it is possible the relationship was in the opposite direction; that is, test counselors who referred more often were more likely to encounter client-level barriers (e.g., adherence concerns) during the referral process. Counselors with more experience might both be more likely to be high referrers *and* anticipate issues related client circumstances, such as adherence, to a greater extent than entry level counselors. Another possibility is that counselors may have faced this client-level referral barrier but had the knowledge and skills to address it and refer. Longitudinal data is needed to evaluate the temporality of these relationships.

Despite the contributions of this study, it had limitations. First, self-reported referral practices are susceptible to bias. Part of the role of an HIV test counselor is to assess and refer to relevant prevention and ancillary services [46]. Given this expectation, counselors may have overestimated their referral rates and underreported referral barriers, particularly those that pertained to their own behaviors. Consistent with this possibility, client-level barriers were endorsed more frequently than counselor-level barriers. Even if counselors were not impacted by social desirability bias, recall bias (i.e., the difficulty of recalling referral rates over a three-month period), may have impacted self-reported referral rates. Future research may benefit from prospectively collecting data on referral practices. Since this study, initiatives have been implemented related to the EHE plan in Miami-Dade County, including organizational tracking of HIV test counselor PrEP referral, which could permit analyses less susceptible to bias. Future studies could leverage organizational data on counselor referral behaviors to prospectively evaluate determinants of referral practices in relation to actual referral practices, as they occur over time. More specifically, as initiatives to improve referral rates are rolled out (e.g., memorandums of understanding from funders of HIV testing services to testing organizations, referral tools to support counselor referral practices), natural experiments could evaluate the degree to which these initiatives are linked to changes in referral rates among counselors. Such data would be useful in identifying evidence-based implementation strategies for improving counselor fidelity to PrEP and behavioral health treatment referral guidelines.

Another challenge of the current study is that because it was designed to be administered in the context of an ongoing training program for HIV test counselors, the research team was advised to design the survey to be as brief as possible. Thus, organizational factors that were not assessed in the current study will be important to evaluate in relation to counselors’ referral practices in future research. For instance, the CFIR guides researchers to evaluate organizational factors that could influence implementation outcomes; although we strived to assess these, additional constructs from the CFIR such as relational connections among team members, communications within organizations, organizational funding and size, and organizational culture would be important to evaluate as potential determinants of referral practices in future studies. Future research should also assess both overall organizational and site-specific information from counselors. In our current study, we collected only the overall organization for which the counselor worked, however in many cases the organization had multiple sites within Miami-Dade County, each of which likely varied with respect to some of these organizational factors (e.g., size, resources, populations served). In addition to between site variation within organizations with multiple locations, counselors may report conflicting statements related to inner setting constructs (e.g. relational connections, organizational structure). Future work will require standardized approaches for handling discrepancies between counselors working within the same site. Future research could explore larger organization and site-specific information for counselors as potential implementation determinants to inform implementation strategy development.

The current study advances knowledge of PrEP and BH referrals. Our integration of a widely used implementation determinant framework (CFIR) can contextualize our findings in the literature. For example, Li and colleagues conducted a systematic review of PrEP implementation determinants, organized within the CFIR [47]. Our findings could be integrated into the dashboard they created because of the common use of the CFIR. Our study also advances the field by focusing on implementer perspectives. A review of HIV implementation research suggested that much has focused on recipient perspectives [47, 48]. Although recipient perspectives are important, implementation requires engagement at multiple levels. By focusing on implementer perspectives in this study, we contribute to knowledge about multilevel determinants that need to be addressed to improve PrEP and BH treatment reach to individuals who may benefit.

## Conclusion

This study contributes to the literature by documenting rates of PrEP and BH referral in an important context, HIV testing. We show that although HIV test counselors surveyed in Miami-Dade County are referring more often than not for PrEP and about half of the time for BH, there are opportunities to increase referrals. Further, our study evaluates the frequency and association of implementation determinants with referral rates. These findings lay the groundwork for developing implementation strategies, tailored to determinants, that could improve referral rates.

## Data Availability

The data (without identifiers) used to support the findings of this study may be made available upon reasonable request (e.g., methodologically sound proposal and signed data use agreement) from the corresponding author following publication. Analyses with this data would only be used to achieve the aims in the approved proposal.

## Footnotes

1 The exception to the compensation was participants from one organization that had a policy against research incentives.
